# Proteomic Analysis of Soybean Roots under Aluminum Stress

**DOI:** 10.1155/2011/282531

**Published:** 2011-03-30

**Authors:** Dechassa Duressa, Khairy Soliman, Robert Taylor, Zachary Senwo

**Affiliations:** Department of Natural Resources and Environmental Sciences, Alabama A&M University, Normal, AL 35762, USA

## Abstract

Toxic levels of aluminum (Al) in acid soils inhibit root growth and cause substantial reduction in yields of Al-sensitive crops. Aluminum-tolerant cultivars detoxify Al through multiple mechanisms that are currently not well understood at genetic and molecular levels. To enhance our understanding of the molecular mechanisms involved in soybean Al tolerance and toxicity, we conducted proteomic analysis of soybean roots under Al stress using a tandem combination of 2-D-DIGE, mass spectrometry, and bioinformatics tools and Al-tolerant (PI 416937) and Al-sensitive (Young) soybean genotypes at 6, 51 or 72 h of Al treatment. Comparison of the protein profile changes revealed that aluminum induced Al tolerance related proteins and enzymes in Al-tolerant PI 416937 but evoked proteins related to general stress response in Al-sensitive Young. Specifically, Al upregulated: malate dehydrogenase, enolase, malate oxidoreductase, and pyruvate dehydrogenase, in PI 416937 but not in Young. These enzymes contribute to increased synthesis of citrate, a key organic acid involved in Al detoxification. We postulate that simultaneous transgenic overexpression of several of these enzymes would be a robust genetic engineering strategy for developing Al-tolerant crops.

## 1. Introduction

Toxic levels of aluminum (Al) in acid soils inhibit root growth and cause substantial reduction in yields of Al-sensitive crops [1, 2]. Its toxicity mechanisms include interference with nutrient and water uptake and translocation [3], disruption of calcium homeostatis [4], disruption of cytoskeleton [5, 6], callose deposition in apoplast that affects movement of substances from cell to cell [7], lipid peroxidation and reactive oxygen species production [8], and interference with cell division and elongation [9, 10]. In concert, these disorders thwart root growth and development that is typically manifested in stunted and swollen root system at the morphological level [11, 12]. 

 Al disrupts cellular components and processes by high binding affinity to phosphate, sulfate, and carbonyl functional groups of cellular components in apoplast and symplast [11]. Perhaps as a direct and parallel evolutionary response to the nature of Al-ligand interaction, plants secret substances that possess these functional groups namely, organic acids [13], phenolics [14–16], and phosphate and polypeptides [17, 18] to bind and detoxify Al in the rhizosphere. Sequestration of Al in the rhizosphere with root secreted organic acids mainly citrate, malate, and oxalate is a common and well-documented physiological mechanism of Al-tolerance in a wide variety of plants [11, 13]. In soybean, Al-tolerance is a quantitatively inherited trait [19] and, physiologically it is correlated with root secretions of citrate [20, 21] and phenolics [16]. 

 Even though the physiological and cellular responses induced by Al stress are direct consequences of change in gene expression and cell metabolism, the genetic components of Al-tolerance pathways are not well understood. Monitoring protein expression changes under Al stress is one possible way of identifying the genetic and molecular components underlying Al-tolerant phenotype. A few research groups have conducted proteome analysis in plant roots under Al stress [22–24]. These studies consistently identified antioxidation and detoxification enzymes and proteins as important determinants of plant Al-tolerance trait. Their limitation, however, is that nonutilized tolerant-sensitive genotype pair that would ensure the specificity of the identified proteins to Al-tolerance mechanism. We previously examined Al-induced protein profile changes in roots of Al-tolerant and Al-sensitive soybean genotypes [16], but did not pursue functional annotation of the differentially expressed proteins. In the present study, we combined 2D DIGE differential expression, mass spectrometry, and bioinformatics tools to characterize the proteome profile changes in Al-tolerant and Al-sensitive soybean genotypes with the aim of identifying potential biomarkers for soybean Al-tolerance. Our results suggest that organic acid biosynthesis and antioxidation and detoxification enzymes play important role in soybean Al-tolerance.

## 2. Materials and Methods

### 2.1. Plant Growth Condition, Root Sampling, and Protein Extraction

Two soybean genotypes PI 416937 (Al-tolerant) and Young (Al-sensitive) were used in the study. Seeds were surface sterilized with 20% household bleach (Clorox) in water for 12 min, rinsed with distilled-deionized water several times and were germinated in standard germination paper at 25°C in an incubator for 72 h. Three-day-old seedlings uniform in tap root length were transferred to black-painted pots filled with approximately 4 L of 800 *μ*M CaCl_2_ background solution with 10 *μ*M Al added (treated) or no Al added (control) in a Conviron growth chamber (16/8 h light/dark cycle with respective temp. of 28°C/20°C, photosynthetic photon density of 100 *μ*mol m^−2^ s^−1^). The pH of the culture solution was adjusted to 4.3 and maintained at that level for the entire duration of the experiment. After 6, 51, or 72 h of Al treatment primary roots and laterals of (see supplementary Figure  1, available online at doi:10.1155/2011/282531, for representative root picture) approximately 15 plants/pot were harvested and immediately flash frozen in liquid nitrogen, stored at −70°C until protein extraction. Protein was extracted using the phenol method [25], and protein concentrations of samples were determined using the Bradford method [26]. 

### 2.2. 2D DIGE Analysis

#### 2.2.1. Sample Preparation and CyDye Labeling

Protein samples were dissolved in 300 *μ*L of 2D cell lysis buffer (30 mM Tris-HCl, pH 8.8, containing 7 M urea, 2 M thiourea and 4% CHAPS) and sonicated at 4°C followed by shaking for 30 min at room temperature. Samples were then centrifuged for 30 min at 14,000 rpm and the supernatant collected. An internal standard for assay normalization was created by mixing equal amounts of protein from each sample. For each sample, 30 *μ*L of protein was mixed with 1.0 *μ*L of diluted CyDye and kept in the dark on ice for 30 min. Proteins from the control and treated samples were labeled with Cy3 and Cy5, respectively, whereas the internal standard was labeled with Cy2. The labeling reaction was stopped by adding 1.0 *μ*L of 10 mM Lysine and incubation in the dark on ice for an additional 15 min. The labeled samples from the control and treated groups and the internal standard were blended for a run of three samples per gel. The 2X 2D sample buffer (8 M urea, 4% CHAPS, 20 mg/mL DTT, 2% pharmalytes, and trace amount of bromophenol blue), 100 *μ*L of destreak solution, and rehydration buffer (7 M urea, 2 M thiourea, 4% CHAPS, 20 mg/mL DTT, 1% pharmalytes, and trace amount of bromophenol blue) were added to the labeling mix to a total volume of 250 *μ*L. The mixture was mixed well and spinned before loading samples into strip holder.

#### 2.2.2. IEF and SDS-PAGE

IEF (pH 3-10 Linear) was run following the protocol of Amersham BioSciences. After IEF, the IPG strips were incubated in fresh equilibration buffer-1 (50 mM Tris-HCl, pH 8.8, containing 6 M urea, 30% glycerol, 2% SDS, trace amount of bromophenol blue and 10 mg/mL DTT) for 15 min with gentle shaking. The strips were then rinsed in fresh equilibration buffer-2 (50 mM Tris-HCl, pH 8.8, containing 6 M urea, 30% glycerol, 2% SDS, trace amount of bromophenol blue and 45 mg/mL DTT) for 10 min with slow shaking. Afterwards, the IPG strips were rinsed with the SDS-gel running buffer and transferred to 12% SDS-gel. SDS-gels were run at 15°C until the dye front began running out of the gel.

#### 2.2.3. Image Scan and Data Analysis

Gel images were scanned immediately following the SDS-PAGE using Typhoon TRIO (Amersham BioSciences). The scanned images were then analyzed by Image Quant software (version 6.0, Amersham BioSciences). The fold changes of protein expression levels were obtained from Biological Variation Analysis (BVA) using the DeCyder software version 6.5 (Amersham BioSciences).

### 2.3. Protein Identification by Mass Spectrometry

#### 2.3.1. Spot Picking, Trypsin Digestion and Mass Spectrometry

The spots of interest were picked by Ettan Spot Picker (Amersham BioSciences) based on the in-gel analysis and spot picking design by DeCyder software. The gel spots were washed few times and digested in-gel with modified porcine trypsin protease (Trypsin Gold, Promega). The digested tryptic peptides were desalted by Zip-tip C18 (Millipore). Peptides were eluted from the Zip-tip with 0.5 *μ*L of matrix solution of cyano-4-hydroxycinnamic acid (5 mg/ml in 50% acetonitrile, 0.1% trifluoroacetic acid, 25 mM ammonium bicarbonate) and spotted on the MALDI plate (model ABI 01-192-6-AB). MALDI-TOF MS and TOF/TOF tandem MS/MS were performed on an ABI 4700 mass spectrometer (Applied Biosystems, Framingham, MA). MALDI-TOF mass spectra were acquired in reflectron positive ion mode, averaging 4000 laser shots per spectrum. TOF/TOF tandem MS fragmentation spectra were acquired averaging 4000 laser shots per fragmentation spectrum on each of the 5 to10 most abundant ions present in each sample, excluding trypsin autolytic peptides and other known background ions.

### 2.4. Database Search

The peptide mass and the associated fragmentation spectra were submitted to GPS explorer workstation equipped with MASCOT search engine (Matrix science) to search the database of National Center for Biotechnology Information non-redundant protein database (NCBInr). Searches were performed without constraining protein molecular weight or isoelectric point, with variable carbamidomethylation of cysteine and oxidation of methionine residues, and with one missed cleavage also allowed in the search parameters. Mass tolerances of 100 ppm for the precursor ion and 0.3 Da for fragment ion were used as constraints to control false positives. Candidates with CI% greater than 95 either for protein or ion score, or *E*-value less than e^−10^ and % identity greater than 30 when blasted against soybean protein database (http://www.phytozome.net/soybean) were considered significant. For peptide data summary and protein prediction significance criteria, see supplementary Tables  1 and  2.

## 3. Results and Discussion

### 3.1. Al-Responsive Proteins Identified By MALDI-TOF-TOF Mass Spectrometry

**Figure 1:**
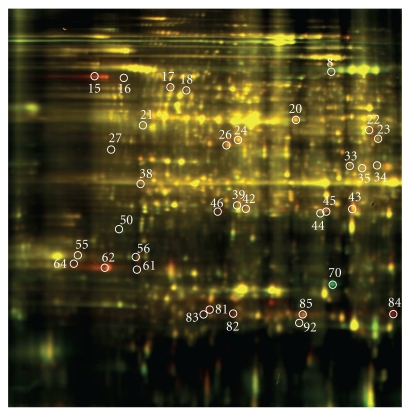
2D profile of aluminum regulated-proteins in PI 416937 72 h posttreatment.

**Figure 2:**
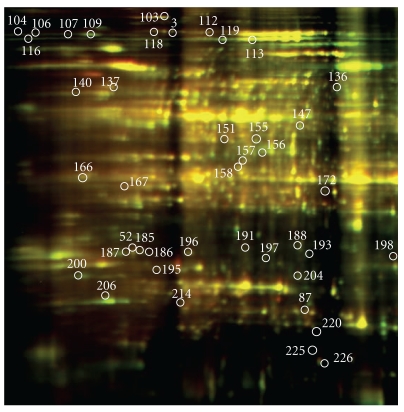
2D profile of aluminum regulated-proteins in Young 72 h posttreatment.

**Figure 3:**
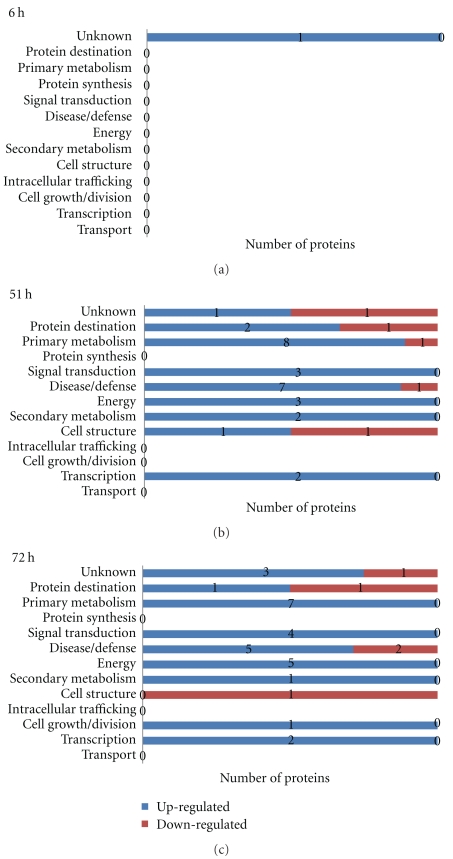
Functional class distribution of aluminum upregulated and downregulated proteins in PI 416937 in time-course experiment.

**Figure 4:**
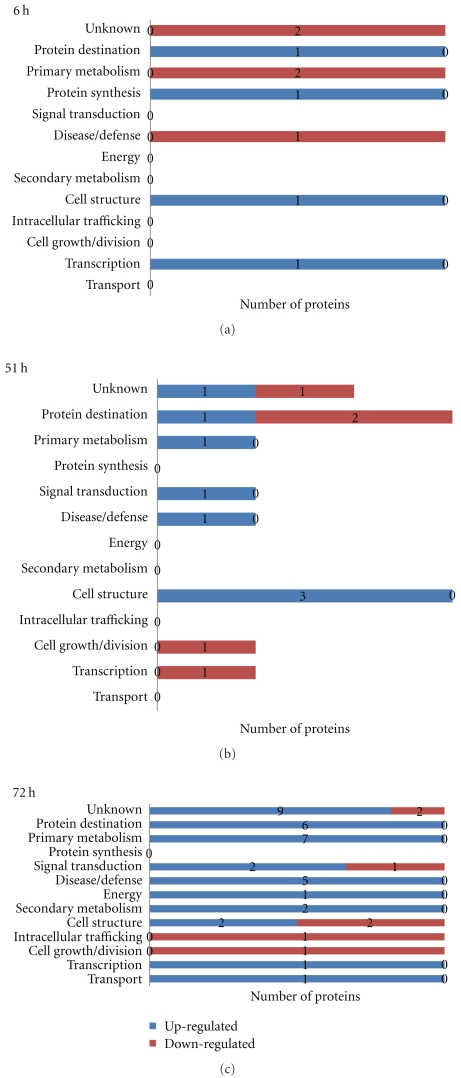
Functional class distribution of aluminum upregulated and downregulated proteins in Young in time-course experiment.

**Figure 5:**
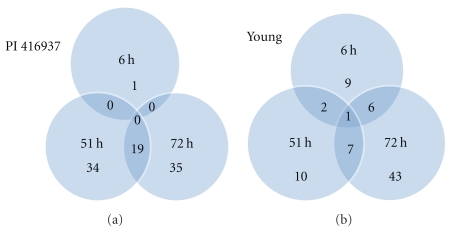
Number of proteins detected at each time points. Numbers in the intersections denote number of proteins in common between or among the time points.

Three criteria were used to identify Al-responsive proteins among hundreds of protein spots resolved by 2-DIGE gel to assure data quality: (1) expression fold change of at least 1.3, either up- or downregulation, at least at one of the time points in Al-treated versus control comparison, (2) presence in all replicates, and (3) standard error less than the mean. A total of 49 proteins in Al-tolerant PI 416937 (Table 1) and 47 proteins in Al-sensitive Young (Table 2) met these criteria and were subsequently identified by mass spec (see Figures 1 and 2, e.g., 2D profile of the identified proteins). In PI 416937, 1, 29, 30 proteins were upregulated at 6, 51, and 72 h, respectively (Figure 3), whereas in Young 4, 7, 36 proteins were upregulated at 6, 51, and 72 h, respectively (Figure 4). Downregulated proteins were 5 each at 51 and 72 h in PI 416937 (Figure 3) and 5, 3, 7 in Young at 6, 51, and 72 h, respectively (Figure 4). In both genotypes, there were more upregulated than downregulated proteins, and more proteins were detected at later stages of Al stress than during the early hours of exposure to Al. There were more common proteins between time points in PI 416937 than in Young (Figure 5).

 In Young, a major change in protein profile was observed 72 h posttreatment, whereas in PI416937 major change in protein expression was observed starting at 51 h of treatment. In addition to the difference in the timing of the molecular response to Al stress between the two genotypes, there was a clear difference in the identity of the expressed proteins as well. Al upregulated several important Al-tolerance proteins (pyruvate dehydrogenase, spot no. 35; malate dehydrogenase, spot no. 42; malate oxidoreductase, spot no.18; and enolase, spot no. 20; cysteine synthase, spot no.39; isoflavone biosynthesis enzyme-NADPH: isoflavone reductase, spot no. 46; dehydroascorbate reductase, spot no. 89) (Table 1), in the Al-tolerant PI 416937. Conversely, aluminum induced mainly non-Al-specific general stress tolerance proteins in the Al-sensitive Young. This might explain the phenotypic difference in Al-tolerance between the two genotypes. Nonetheless, Vacoular ATpase (V-ATPase)—a known Al-tolerance transport protein (spot no. 155, Table 2) was upregulated 1.3-fold at 72 h in Young indicating that Young may possess some basal tolerance mechanism to aluminum toxicity as suggested by Bianchi-Hall et al. [19].

### 3.2. Functional Classification of Al-Responsive Proteins

The identified proteins were grouped into 13 classes based on cellular function according to the classification scheme in Houston et al. [27]. The distribution of the proteins over functional classes revealed that Al-responsive proteins in PI 416937 are more of proteins of primary metabolism, disease/defense, energy and signal transduction with 16% in the unknown class (Figure 3); and in Young Al-responsive proteins are more of proteins of primary metabolism, disease/defense and protein destination with 26% in the unknown class (Figure 4). In discussion of this paper, the disease/defense category was renamed as antioxidation and detoxification and the energy category as organic acid biosynthesis enzymes to easily connect these proteins to physiologically known plant Al-tolerance mechanisms.

### 3.3. Antioxidation and Detoxification Proteins

Al toxicity induces production of reactive oxygen species (ROS) in cells beyond the level generated by normal metabolism as one mode of its toxicity [8, 12, 23]. Plants have elaborate enzymatic and nonenzymatic mechanisms for ROS scavenging and variation in plant antioxidant capacity has been shown to correlate with variation in plant Al-tolerance level [28]. Several known antioxidation and detoxification enzymes including thioredoxin (Spot 15), lactoyl glutathione lyase (spot 81), three isoforms of glutathione-S-transferase (GST) (spots 75, 82, and 85), dehydroascorbate reductase (spot 89), cysteine synthase (spot 39), aldo/keto reductase (spot 43) and NADH: isoflavone reductase (spot 46) were upregulated in Al-tolerant PI 416937 (Table 1). None of these enzymes except glutathione-S-transferase (GST) was upregulated in Al-sensitive Young. Earlier proteome analysis in Al-tolerant varieties of rice [22], soybean [24], and tomato [12, 23] suggest that antioxidation and detoxification enzymes play an important role in internal Al-tolerance mechanism. PI 416937 accumulates less Al than Young in symplast [29] and, as revealed by the current study, appears to have better antioxidant and detoxification capacity implying that it employs both the exclusion and internal detoxification mechanisms of Al-tolerance. 

 Among the antioxidation and detoxification enzymes differentially expressed between PI 416937 and Young, it is interesting to highlight the Al detoxification role of cysteine synthase, GST, and NADPH: isoflavone reductase. There is increasing evidence for the synergetic effect of cysteine synthase and GST in conferring plant Al-tolerance [12, 22, 24]. Cysteine, the byproduct of cysteine synthase catalyzed reaction, is a precursor for glutathione synthesis (GSH) which in turn is a key substrate for biosynthesis of metallothionin (MT) and phytochelatin- (PC-) low molecular weight peptides known to confer heavy metal tolerance in plants [30]. MT has been reported to be induced by Al stress probably as a tolerance mechanism [24]. In addition to its role in MT and PC biosynthesis, GSH is directly involved in detoxification of Al-elicited toxins in concert with GST. GST transfers GSH to such substances forming s-gluthionaylated reaction products that are nontoxic to the cell [31]. Further, reduced glutathione has an antioxidant property capable of scavenging Al-triggered ROS. Therefore, the concomitant upregulation of cysteine synthase and glutathione-S-transferases in Al-tolerant PI416937 is suggestive of the significance of antioxidation and detoxification proteins in protecting this soybean type from Al-induced oxidative stress and endogenous toxins. NADPH: isoflavone reductase is involved in isoflavone synthesis. Flavonoids confer Al-tolerance via the dual mechanisms of Al chelation and antioxidation [18]. At the physiological level, a positive correlation between root flavonoids and phenolics secretion and plant Al-tolerance has been documented [14–16].

### 3.4. Cell Structure and Cell Division-Related Proteins

Plant cells require dynamic actin- and tubulin-based networks for proliferation and differentiation [32]. Aluminum stabilizes, depolymerizes, and alters the orientation of cytoskeleton networks resulting in inhibition of cell division, elongation and consequently root growth [5, 6, 33, 34]. Whether this phenomenon is a direct outcome of Al-interaction with cytoskeletal structure or by its indirect influences on factors that control cytoskeletal assembly and disassembly is not known [6]. The expression of cytoskeleton proteins has been proposed to be one of the primary targets of Al toxicity [33]. Al-induced increase in abundance of mRNA level for actin-bundling/rigidifying protein fimbrin [35] and actin protein [12] has been reported. In the present study, actin protein was upregulated at 51 h of treatment in Al-tolerant PI 416937, whereas alpha tubulin was downregulated at 51 and 72 h (Table 1). In Al-sensitive Young, alpha tubulin was upregulated at 51 h and downregulated at 72 h and tubulin beta-3 was upregulated at 72 h (Table 2). These results show that the two genotypes differ in expression of cytoskeletal proteins under Al stress. However, how this dynamics relates to Al-induced cytoskeleton stabilization and reorientation that leads to root growth inhibition is yet to be determined. It has been shown that Al-induces mechanical rigor/stability in actin networks of soybean root cells [33]. Thus, the upregulation of actin protein in Al-tolerant PI 416937 could be an adaptive response to Al stress. New actin networks could form from newly synthesized actin protein and compensate for networks stabilized by Al. Apart from cytoskeleton proteins, one important protein related to cell division, BRAC1, was upregulated in PI 416937 at 72 h of treatment. BRAC1 is a DNA damage and cell cycle control protein and its homolog is a breast cancer inhibitor in human [36]. DNA damage is one mechanism of Al mediated cellular injury [18], suggesting BRAC1 might be an important novel candidate biomarker for plant Al-tolerance.

### 3.5. Organic Acids Biosynthesis Enzymes

**Figure 6:**
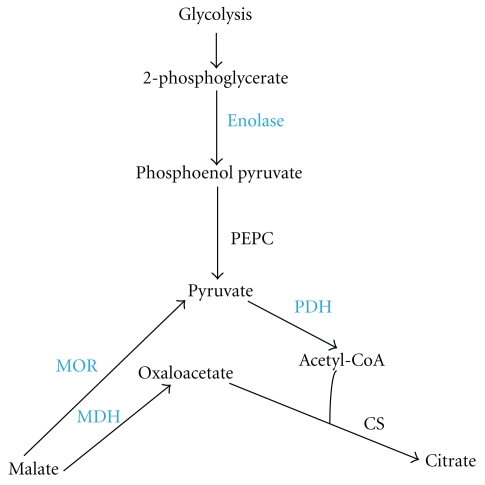
Diagrammatic representation of enzymes involved in citrate synthesis upregulated in Al-tolerant PI 416937. Enzymes in blue color are those upregulated. PEPC: phosphoenol pyruvate carboxylase; PDH: pyruvate dehydrogenase; CS: citrate synthase; MOR: malate oxidoreductase; MDH: malate dehydrogenase.

 In response to Al stress, root accumulation and secretion of organic acids increase particularly in Al-tolerant plant genotypes [21, 37–39], which in most cases is preceded by an increase in expression and activities of enzymes involved in their biosynthesis [12, 39]. However, the particular TCA cycle enzyme(s) activated by Al and the organic acid anions modulated are species specific [39, 40]. For example,in Al-tolerant microbe (*Pseudomonas fluorescens) *that depends on oxalate for Al detoxification, Al reconfigures the enzymatic reactions of the TCA cycle in favor of the synthesis of glyoxylate-a precursor for oxalate, by upregulating isocitrate layse and downregulating isocitrate dehydrogenase and *α*ketoglutarate dehydrogenase complex [40]. At the physiological level, soybean Al-tolerance is correlated with increased levels of tissue citrate accumulation and secretion [20, 21]. Nonetheless, the specific TCA cycle enzymes that modulate organic acid metabolism in soybean in response to Al stress have not been identified. Here, we provide the first evidence at protein expression level that show Al simultaneously induces enolase 1 (spot 20), malate dehydrogenase (spot 42), malate oxidoreductase (spot 18), and pyruvate dehydrogenase E1 (spot 35) (Table 1), in roots of Al-tolerant soybean PI 416937. None of these enzymes was triggered by Al in the Al-sensitive Young underscoring the specificity of the response to Al-tolerance mechanism. All of these enzymes contribute to citrate biogenesis (Figure 6), which could explain the increased citrate accumulation and secretion observed in Al-tolerant PI 416937 under Al stress by Silva et al. [21]. 

 Engineering enzymes of the TCA cycle have been the focus of one aspect of plant Al-tolerance research for over a decade. Transgenic upregulation of individual TCA cycle enzymes such as malate dehydrogenase, citrate synthase and phosphoenolpyruvate carboxylase (PEPC) have had some success in producing transgenic plants with enhanced Al-tolerance [41–44]. We speculate that concomitant over-expression of several of these enzymes, as exemplified by natural plant defense response in our study, might be a plausible genetic engineering strategy for developing transgenic crops adapted to high Al soils. A net increase in cellular citrate level is a balance of its formation and degradation. Therefore, transgenic downregulation of enzymes involved in its breakdown is an alternative scenario for improving plant Al-tolerance. While there is no experimental data available regarding how such transgenic plants would perform under nonstressful environment, improved Al-tolerance level has been observed in yeast mutants null for citrate degrading enzymes [45]. Also in Al-tolerant maize, Piñeros et al. [46] observed Al-induced reduction in the activity of isocitrate dehydrogenase (a citrate degrading enzyme), which paralleled an increase in root citrate level.

### 3.6. Signal Transduction and Gene Regulation Proteins

Perception of stress signal and subsequent changes in gene expression, cell metabolism, and physiology are key events in plant adaptation response to a stress factor. Al-signaling pathway has not been elucidated yet with the exception of the identification of one protein (cell wall associated kinase 1, WAK 1) as a possible component of Al signal transduction pathway [47]. Serine/theorine protein kinase and protein phosphatase 2A, both with signaling function, were identified in the present study. Both were upregulated in Al-tolerant PI 416937 and serine/theorine kinase in Al-sensitive Young. Earlier proteome analysis studies suggest that G-protein or GTP binding protein is involved in Al signaling [22, 24]. No change in the expression level of this protein was observed in the present study. However, the activity of G-protein coupled receptor is controlled by its phosphorylation state, which in turn is attenuated by protein kinase and phosphatase. The upregulation of serine/theorine protein kinase and protein phosphatase 2A might therefore have relevance in Al stress signaling. ROS signaling 14-3-3-like protein (spots 55 & 64) was upregulated in PI416937, but downregulated in Young. One transcription factor CREB binding (cAMP element binding) protein (spot 23) was upregulated in PI 416937. CREB binds to CRE (cAMP responsive element) and regulates the transcription of several genes. It modulates several physiological functions including brain memory in human [48], but its role in plant system has not been well documented. Nevertheless, the fact that it regulates the expression of several genes makes it an interesting putative gene for Al stress tolerance. 

### 3.7. Chaperone and Protease Proteins

 Several chaperones, protease, and proteasome subunit proteins were upregulated in both genotypes. These proteins are crucial for survival of biota under adverse conditions and respond to various biotic and abiotic stress factors including Al [24]. They play a role in protein folding, translocation, degradation, and assembly both under normal and stress conditions [49]. Stress conditions increase the activity of these proteins by altering the intricate balances in cellular protein synthesis and turnover.

## 4. Conclusion

Aluminum induced a distinct protein profile changes in Al-tolerant and Al-sensitive soybean genotypes. The Al-tolerant genotype PI 416937 expressed multitude of Al-tolerance-specific proteins when mildly intoxicated with Al, whereas the sensitive genotype Young lacks such Al specific response. The comparative proteome analysis enabled us to identify two enzyme categories namely, enzymes of the antioxidation and detoxification systems and organic acids biosynthesis pathway as important players in soybean Al-tolerance. We also identified two novel proteins, BRAC1: a DNA damage control and cell cycle regulator, and CREB: transcription factor, which might contribute to soybean adaptation mechanisms to Al toxicity. Increased organic acid synthesis, accumulation, and root secretion are the well-known physiological mechanisms of plant Al-tolerance. At the molecular level, attempts to engineer Al-tolerant plants by modulating the activities of individual organic acid biosynthesis enzymes have produced encouraging results [41–44]. In the present study, we observed a concurrent upregulation of several organic acid biosynthesis enzymes in Al-tolerant soybean PI 416937 in response to Al stress which suggests that simultaneous transgenic over-expression of these enzymes might be a more robust genetic engineering strategy for developing Al-tolerant plants than the engineering one enzyme at a time paradigm.

## Figures and Tables

**Table 1:** Aluminum regulated-proteins detected in soybean line PI 416937 in time-course Al stress experiment.

Spot	Accession	Protein ID/functional class^a^	Average expression fold change^b^					
			6 h	SE	51 h	SE	72 h	SE
5	gi/126411	*Lipoxygenase (PM)*	−1.04	0	−1.43	0.09	−1.25	0.07
8	gi/42820320	*Copper amino oxidase (DD)*	−1.04	1.05	−1.53	0.02	−2.24	0.08
15	gi/225425555	*Thioredoxin (DD)*	1.11	0.02	3.01	0.07	4.34	1.04
16	gi/224115920	*Protein phosphatase 2A (ST)*	1.13	0	1.83	0.14	3.05	0.4
17	gi/22331670	*Serine/threonine protein kinase (ST)*	−1.24	0.12	1.14	0.06	1.47	0.05
18	gi/15225438	*Malate oxidoreductase (E)*	−1.13	0.07	1.10	0.04	1.33	0.04
20	gi/162458207	*Enolase (E)*	1.07	0.04	1.04	1.12	1.39	0.03
21	gi/223548395	*26S protease subunit 6a (PD)*	−1.13	0.01	−1.29	0.04	−1.31	0.19
22	gi/2511541	*DNA-binding protein gbp16 (UN)*	1.03	—	1.48	0.21	1.90	0.23
23	gi/4836923	*CREB binding protein (TF)*	−1.05	0.03	−1.08	1.21	1.38	0.14
24	gi/223548531	*S-adenosyl methionine synthase (PM)*	1.13	1.17	1.41	0.35	1.45	0.14
25	gi/224285989	*Amidase/tetratricopeptide repeat (PM)*	−1.08	0.14	1.62	0.07	nd	—
26	gi/3024127	*S-adenosyl methionine synthase (PM)*	1.11	0.06	1.35	0.23	1.50	0.26
27	gi/223548639	*Transaldolase (PM)*	1.07	0.02	1.16	0.01	1.43	0.14
30	gi/211970690	*Formate dehydrogenase (PM)*	1.14	0.01	1.45	0.17	1.22	0.21
31	gi/1498340	*Actin (CS)*	1.06	0.02	1.35	0	1.05	1.37
33	gi/157346459	*NADH:flavin oxidoreductase (DD)*	1.05	1.08	1.49	0.09	1.30	0.1
34	gi/242462	*Lipoxygenase (PM)*	nd	—	1.71	0.08	1.62	0.3
35	gi/195637880	*Pyruvate dehydrogenase E1 (E)*	1.02	1.03	1.30	0.04	1.30	0.01
36	gi/9230771	*Cyt phosphoglysrate kinase (PM)*	nd	—	1.59	0.21	1.27	0.11
37	gi/49257111	*Disulfide isomerase-like (PD)*	−1.12	1.26	−1.63	0.02	1.02	1.32
38	gi/168029670	*Unknown (UN)*	nd	—	nd	—	1.61	0.38
39	gi/126508778	*Cystine synthase (PM)*	1.08	0.04	1.40	0.16	1.46	0.16
42	gi/3273828	*Malate dehydrogenase (E)*	1.03	1.1	1.87	0.55	2.05	0.62
43	gi/186506243	*Aaldo/keto reductase (PM)*	1.14	0.06	1.52	0.1	1.48	0.27
44	gi/34559418	*(+)-pulegone reductase (SM)*	1.09	1.15	1.31	0.24	1.43	0.26
45	gi/108864466	*Unknown (UN)*	nd	—	1.64	0	1.85	0.09
46	gi/2687724	*NADPH:isoflavone reductase (SM)*	1.24	0.22	nd	—	2.17	0.33
50	gi/393401	*Alpha tubulin (CS)*	1.09	0.07	−1.45	0.05	−1.30	0.09
55	gi/3023196	*14-3-like protein C (ST)*	nd	—	nd	—	1.41	0.27
56	gi/6469121	*Plasma membrane polypeptide (UN)*	nd	—	nd	—	1.30	0.07
61	gi/186507172	*BRCA1 C terminal (CD)*	1.09	0.04	1.21	0.04	1.41	0.01
62	gi/6690745	*Resistance protein (DD)*	nd	—	nd	—	3.81	0.04
64	gi/3023194	*14-3-3-like protein A (ST)*	1.11	0.08	−1.15	0.06	1.60	2.11
65	gi/125550993	*Unknown (UN)*	1.00	1.01	−1.48	0.02	−1.28	0
67	gi/225455804	*Hypothetical protein (UN)*	1.04	1.09	1.61	0.12	1.22	0.03
70	gi/226866	*31 kD protein (UN)*	2.19	0.67	−1.28	1.81	−1.31	4.14
75	gi/11385431	*Glutathione S-transferase 8 (DD)*	−1.04	1.06	1.39	0.07	1.16	1.17
78	gi/224087343	*WD40 protein/RNA processing (TF)*	−1.06	1.15	1.34	0.15	1.18	0.02
79	gi/194705252	*Cytidyly transferase (SM)*	−1.25	0.08	1.64	0.24	1.00	0.9
81	gi/1170781	*Glutathatione-S-transferase 3 (DD)*	1.10	1.64	4.05	0.92	2.27	2.03
82	gi/11385435	*Glutathione-S-transferase 10 (DD)*	1.12	1.23	2.30	0.16	1.63	0.75
83	gi/399240	*20 kDa chaperonin (UN)*	−1.02	1.39	2.29	0.33	1.54	2.14
84	gi/224135489	*Beta glucosidase (PM)*	1.16	0.03	nd	—	4.02	0.48
85	gi/11385459	*Gluthstione-S-transferase 22 (DD)*	−1.07	1.16	1.55	0.01	1.59	0.26
89	gi/28192427	*Dehydroascorbate reductase (DD)*	1.09	0.049	1.35	0.05	−1.08	1.62
90	gi/30682123	*Adenine phosphoribosyl transferase, ATP4 (PM)*	1.24	0.01	1.38	0.02	nd	—
91	gi/17380185	*Proteasome subunit beta-1 (PD)*	−1.07	1.16	1.42	0.14	nd	—
92	gi/21068664	*Quinine oxidoreductase (DD)*	1.21	0.01	−1.20	0.14	−1.31	0.14

^a^PM: primary metabolism, SM: secondary metabolism, E: energy, PD: protein destination, TF: transcription factor, CS: cell structure, ST: signal transduction, DD: disease/defense, CD: cell division, UN: unknown function;

^b^expression fold change at each time point, negative values: downregulation, positive values: upregulation, fold change significance cut-off is ±1.3, SE: standard error, nd: not detected.

**Table 2:** Aluminum regulated-proteins detected in soybean cultivar Young in time-course Al stress Experiment.

Spot	Accession	Protein ID/functional class^a^	Average expression fold change^b^					
			6 h	SE	51 h	SE	72 h	SE
3	gi/27883937	*Phosphoribosylformayl glycinamidine synthase (PM)*	−1.34	0.13	1.33	0.10	1.45	0.20
52	gi/224160640	*Zinc Knuckle (UN)*	1.24	0.18	nd	0.17	1.67	0.17
87	gi/11385431	*Glutathione (DD)*	nd	—	1.22	0.08	4.34	1.04
100	gi/156938901	*Profilin/actin binding (CS)*	1.92	0.8	nd	—	nd	—
103	gi/79321519	*Heat shock protein 70 (PD)*	−1.02	1.10	1.08	1.10	1.44	0.06
104	gi/153805634	*Unknown (UN)*	nd	—	nd	—	1.38	0
106	gi/15219879	*Apoptotic ATpase (DD)*	−1.08	0.01	1.15	0.11	1.44	0.10
107	gi/30696901	*Xylose isomerase (PM)*	1.05	0.01	nd	—	1.37	0.09
109	gi/168030956	*Serine/threonine*∖*protein kinase (ST)*	nd	—	nd	—	1.31	0.04
112	gi/436169	*Lipoxygenase (PM)*	−1.10	0.03	1.13	0.05	1.35	0.23
113	gi/77552532	*Unknown (UN)*	−1.52	0.62	1.21	0.06	1.38	0.18
116	gi/2232254	*NADH:flavin oxidoreductase (UN)*	−1.36	0.06	nd		1.49	0.18
118	gi/223541365	*Transitional endoplasmic reticulum ATPase (CS)*	nd	—	1.37	0.03	1.37	0.27
119	gi/223974443	*Unknown (UN)*	−1.05	0.01	−1.03	1.07	1.31	0
133	gi/42820320	*Copper amino oxidase (DD)*	1.03	1.05	nd	—	1.35	0.07
136	gi/195636596	*T-complex protein 1 alpha (PD)*	1.06	0.02	1.35	0	1.05	1.37
137	gi/125574688	*Glutathione-s-transferase (DD)*	1.19	0.17	1.21	0.08	1.33	0.35
140	gi/74053562	*Tubulin beta-3-chain (CS)*	−1.03	0.01	1.02	0.08	1.62	0.3
147	gi/5225811	*Unknown (UN)*	1.03	0.03	1.05	1.06	−1.39	0.01
151	gi/124112056	*Septum site determining protein (CS)*	nd	—	1.52	0.53	−1.36	0.05
155	gi/87240711	*V-ATPase subunit C (DD)*	nd	—	nd	—	1.31	0.23
156	gi/1346698	*Phosphoglycerate kinase (PM)*	−1.03	0	1.14	1.20	1.45	0.54
157	gi/1582580	*Caffeic acid O-methyl transferase (SM)*	−1.04	1.06	nd	—	1.35	0.09
158	gi/1346028	*Polyprenyl synthetase (SM)*	1.09	1.09	1.06	0.04	1.32	0.05
166	gi/3023197	*14-3-like (ST)*	nd	—	1.09	0.04	−1.32	0.23
167	gi/393401	*Alpha tubulin (CS)*	1.21	0	1.41	0.06	−1.42	0.20
172	gi/78146198	*MADS box protein/transcription factor (TF)*	1.11	0.11	nd	—	1.85	0.74
185	gi/115511406	*Acirduyctase dioxygenase (PM)*	1.25	0.13	nd	—	1.61	0.01
186	gi/224105487	*Unknown (UN)*	1.11	1.22	1.22	0.21	1.34	0
187	gi/145352433	*Unknown (UN)*	−1.01	1.02	1.44	0.01	1.59	0.25
188	gi/17380185	*Proteasome subunit beta type-1 (PD)*	1.04	0.02	nd	—	1.72	0.11
191	gi/223543735	*Proteasome subunit beta type (PD)*	−1.04	0.02	nd	—	1.74	0.52
193	gi/145340582	*Unknown (UN)*	1.08	0	nd	—	1.41	0.08
195	gi/209778987	*Putative rab1C protein (IT)*	−1.05	1.15	−1.02	1.10	−1.67	0.29
196	gi/18395025	*20S proteasome beta subunit C (PD)*	1.07	1.16	nd	—	1.32	0.27
197	gi/125577605	*DNJ/HSP40 (DD)*	1.04	1.16	1.00	1.07	1.35	0.31
198	gi/34485411	*Resistance protein (DD)*	1.00	1	1.40	0.06	1.73	0.37
200	gi/20140683	*Translationally-controlled tumor protein homolog (CD)*	nd	—	−1.53	0.08	−1.46	0.10
201	gi/40644130	*Allene oxide cyclase (PM)*	−1.51	0.47	nd	—	1.59	0.26
204	gi/223510245	*Unknown (UN)*	1.04	0.04	1.01	1.17	1.32	0.05
206	gi/197294157	*Hypothetical protein (UN)*	−1.53	0.55	nd	—	1.42	0.22
212	gi/110931690	*MYB transcription factor (TF)*	1.39	0.38	−1.93	0.75	nd	—
214	gi/18143656	*Mcp20 (PD)*	1.35	0.18	nd	—	1.61	0.73
218	gi/829282	*Eukaryotic initiation factor A (PS)*	1.93	0.46	nd	—	nd	—
220	gi/15238219	*Unknown (UN)*	nd	—	−1.32	0.08	−1.72	0.08
225	gi/223547693	*Electron transporter (E)*	nd	—	−1.02	1.07	2.10	0.20
226	gi/145345251	*Glycogen synthase kinase-3 (PM)*	nd	—	nd	—	1.77	0.21

^a^PM: primary metabolism, SM: secondary metabolism, E: energy, PD: protein destination, TF: transcription factor, CS: cell structure, ST: signal transduction, DD: disease/defense, t, IT: intracellular trafficking, CD: cell division, UN: unknown function;

^b^expression fold change at each time point, negative values: downregulation, positive values: upregulation, fold change significance cut-off is ±1.3, SE: standard error, nd: not detected.

## Abbreviations

2D DIGE: two-dimensional differential in gel electrophoresis
IEF: isoelctro focusing
MALDI-TOF MS: Matrix-assisted laser desorption ionization-time of flight mass spectrometry
IPG: Immobilized pH gradient
SDS-PAGE: Sodium dodecyl sulfate polyacrylamide gel electrophoresis
DTT: Dithiothreitol
CHAPS: 3-[(3-Cholamidopropyl) dimethylammonio]-1-propanesulfonate
ROS: Reactive oxygen species
BRAC1: Breast cancer 1 gene
CREB: cAMP response element-binding transcription factor
CI%: Confidence interval percentage
TCA cycle: Tricarboxylic acid cycle.
